# How Tobacco Hurts

**Published:** 1875-06

**Authors:** 


### HOW TOBACCO HURTS.

The papers are making lots of sport over the fact that some doctor has said that "the use of tobacco interferes with the molecular changes coincident with the development of the tissues, and makes the blood-corpuscles oval and irregular at the edge." One funny fellow declares that he wouldn't have the edges of his blood-corpuscles made oval and irregular for the world. It would be bad enough, he thinks, to have them made regularly oval, but anything like irregularity in their form he could not well endure.

It is all very well to laugh at the professional language employed,—language which sounds stilted and unnatural to laymen, but when we tell our friends that to interfere with "molecular changes" which must occur in order to supply wasted tissues, is simply to secure degeneration of one kind or another in the whole system, and that to alter essentially the form of the "blood-corpuscles" is to retard the circulation of the vital fluid and to lower the tone of the system; to render the constitution quite powerless to ward off disease or do anything like successful battle with the enemies in the air and all about us—we trust, we render it more apparent that the fact is not a thing to be laughed at, however it may be with the language.

But tobacco hurts in other ways besides interferig with the circulation of the blood. Its primary influence is exerted upon the brain. Here it works with marvellous facility. As a brain-stimulant and poison it takes the premium, because it strikes deep and its effects linger long. Let a boy of 15 years, use tobacco energetically for one year, and he can never recover wholly from its poisonous effects, though he live to old age. By his year of folly he has materially shortened his days.

Besides this, he has lessened his power for usefulness by drawing largely upon his supply of vitality. If exposed to disease of a contagious character, he will be likely to take it, because his system lacks the vitality to ward it off. His year of folly and poisoning has, furthermore, changed his stomach from a wonderful and perfect instrument, to a disorganized and troublesome appendage. Food, which was formerly rapidly and comfortably transformed into warmth and blood, and tissue, now sours and "sets heavy" on the demoralized stomach; and, in short, the wonderful machinery of life is all out of order.

The heart goes wrong, too. It has gone on, contracting and expanding, at the rate of over one hundred thousand times every day, but as the form of each separate atom of blood changes, from the effects of the poison, it finds its labor greatly increased. The blood which it is expected to pump through the little long tubes, moves slowly, for the friction is great. So the pumping machine falters and halts, occasionally. Again, it will pump a few strokes with greatly increased rapidity, though not with natural power. This is bad for the pump, *very* bad for the individual. It is called "heart-disease"—a disease which can be secured, unfailingly, by the use of tobacco. All tobacco-users have it more or less, and it kills several thousand Americans every year.

One thing more. Would any of our young readers be willing to engage in an employment or amusement which rendered them unfit to live in a house, and which should consign them to a stable? Would any decent person be willing to incur the charge of being absolutely unfit to live with a clean horse or cow, by reason of some filthy habit? If it would annoy them to feel that they could by any possibility, be too dirty to live with cattle, let us say to them that he who saturates his clothing and his body with disgusting and poisonous tobacco, makes a thorough nuisance of himself, does a great wrong to himself and to all with whom he comes in contact, and positively insults the horse or the cow which he approa[illegible]hes. The time will come when men and boys who do these vile things, will be compelled to fly to some hiding-place to indulge in their disgusting practices, which will be placed under a ban, precisely as is the less destructive leprosy in the East, or small-pox with us.

